# Effect of Directional Deep Brain Stimulation on Sensory Thresholds in Parkinson’s Disease

**DOI:** 10.3389/fnhum.2020.00217

**Published:** 2020-06-09

**Authors:** Shelby Sabourin, Olga Khazen, Marisa DiMarzio, Michael D. Staudt, Lucian Williams, Michael Gillogly, Jennifer Durphy, Era K. Hanspal, Octavian R. Adam, Julie G. Pilitsis

**Affiliations:** ^1^Department of Neuroscience and Experimental Therapeutics, Albany Medical College, Albany, NY, United States; ^2^Department of Neurosurgery, Albany Medical College, Albany, NY, United States; ^3^Department of Neurology, Albany Medical College, Albany, NY, United States

**Keywords:** deep brain stimulation, Parkinson’s disease, directionality, mechanical thresholds, sensory thresholds

## Abstract

**Objective:**

Previous studies showed that deep brain stimulation (DBS) relieves pain symptoms in Parkinson disease (PD) patients when programmed for motor-symptom relief. One factor involved in pain processing is sensory perception of stimuli. With the advent of directional leads, we explore whether directional DBS affects quantitative sensory testing (QST) metrics acutely.

**Methods:**

PD patients with subthalamic (STN) DBS and directional leads were tested in 5 settings (DBS-OFF, DBS-ON with omnidirectional stimulation, and DBS-ON) for each of three directional segments of contact used for clinical programming. The Unified Parkinson’s Disease Rating Scale (UPDRS-III) assessed patient’s motor skills at time of study visit at clinical contact and at contact which produced optimal sensory threshold (defined by the greatest tolerance to mechanical stimuli). Correlation analyses were performed between stimulation parameters [amplitude, frequency, pulse width (PW), total electrical energy delivered (TEED)] and outcome metrics.

**Results:**

Sensory thresholds were obtained in nine patients. Directional stimulation did not significantly alter patient perceptions of sensory stimulus [cold pain (*p* = 0.69), warm pain (*p* = 0.99), Von frey fibers (*p* = 0.09), pin-prick (*p* = 0.88), vibration (*p* = 0.40), pressure (*p* = 0.98)]. With correlation analysis, increasing PW at the posterior contact increased pin prick and vibration sensitivity (*p* < 0.001). Additionally, an increase in TEED caused a decrease in sensitivity to warm detection when using the anterior (*p* = 0.04), lateral (*p* = 0.02), and medial contacts (*p* = 0.03), and also caused a decrease in sensitivity to cold detection when using the medial contact (*p* = 0.03). UPDRS-III remained stable during testing.

**Conclusion:**

Motor benefit can be acutely maintained at directional contacts, whereas directional stimulation can modulate thermal and mechanical sensitivity. Further investigation will determine whether these changes are maintained chronically or can be improved with optimized programming.

## Introduction

Parkinson’s disease (PD) is a debilitating neurodegenerative disorder that affects over 10 million people worldwide Parkinsons Foundation (2019)^1^. The progressive nature of the condition can result in severe motor impairments that could lead to a patient’s loss of independence. The prevalence and severity accounts for a $52 billion USD burden on our healthcare system Parkinsons Foundation (2019)^1^. Early in diagnosis, levodopa is often prescribed in conjunction with carbidopa (termed L-DOPA) to replenish depleted dopamine supplies in the striatum (LeWitt, 2015; Tarakad and Jankovic, 2017). When patients become refractory to medical management, deep brain stimulation (DBS) of the subthalamic nucleus (STN) is a well-established neuromodulatory treatment shown to improve the motor symptoms of PD (Limousin et al., 1998). In addition, this treatment may improve non-motor symptoms in PD patients. Pain is on such symptom and in pre-clinical studies, quantitative sensory testing (QST) is a method of measuring sensory thresholds that may be used as a surrogate. Previously both our laboratory and others have examined sensory thresholds in PD patients with DBS. Preliminary research of sensory thresholds noted that thermal perception was worse in PD patients not receiving stimulation as compared to healthy controls, but also found that this thermal perception improved in PD patients once stimulation was turned on (Maruo et al., 2011). In PD patients also experiencing chronic pain, low frequency stimulation (LFS) was found to produce lower heat thresholds compared to both high frequency stimulation (HFS) and no stimulation (Belasen et al., 2017). The study further found that patients had increased detection of vibration and mechanical pressure when receiving LFS as compared to HFS. These indications show potential for the use of DBS to modulate sensory disturbances in PD patients.

The use of directional leads, which alter current distribution to more precisely target the STN, is a novel addition to DBS which has resulted in larger therapeutic windows and higher side-effect thresholds while maintaining improvements in motor function (Pollo et al., 2014; Steigerwald et al., 2016; Dembek et al., 2017). However, there has been no investigation as to the effect of directional DBS (dDBS) on sensory thresholds of PD patients. In this study, we aim to investigate the improvement in sensory perception of PD patients treated with dDBS. Elucidating the effect of this treatment on sensory thresholds would aid in improving the quality of life of patients suffering from PD and expanding the knowledge of treatment strategies for the disease.

## Methods

### Participants

In this study, participants were considered eligible if they had previously been diagnosed with PD and had undergone standard of care implantation of bilateral or left-sided directional STN DBS lead(s). Those who qualified for surgical treatment had completed the Unified Parkinson’s Disease Rating Scale motor scale (UPDRS-III) and neuropsychological testing as part of the standard unit of care. Patients must have had improvement of more than 30% on the Core Assessment Program for Surgical Interventional Therapies (CAPSIT) to be an eligible candidate for surgery. Patients who demonstrated dementia, significant cognitive impairment, or unstable psychiatric disease were considered ineligible. Subjects enrolled in this approved study were fluent in English and competent to consent to participation.

### Procedure

Patients completed questionnaires at the start of the visit to gauge the pain level of the patient, assess sensitivity to daily activities, and assess their perception of specific pain types at their clinical settings. The questionnaires included the Numerical Rating Scale (NRS) and the King’s Parkinson’s Disease Pain scale (KPDPS). Using NRS, patients were asked to rate (1) pain at its worst during the previous week, (2) pain at its best during the previous week, (3) pain on average, and (4) current pain. Using KPDPS, patients were asked to rate their severity (0-3) and frequency (0-4) of their pain in (1) musculoskeletal pain, (2) chronic pain, (3) fluctuation-related pain, (4) nocturnal pain, (5) oro-facial pain, (6) discoloration, and (7) radicular pain all in the last month as it related to PD. Additionally, the location and history of a patient’s primary source of pain was recorded prior to sensory testing.

The participant then went through a series of QSTs for about 1–1 1/2 h at different settings to assess detection and pain thresholds for mechanical and sensory stimuli. Only the left lead was tested based on anecdotal evidence in our patient population that left sided implants had greater pain responses. Testing one lead also controlled for heterogeneity and the confounding factor of bilateral stimulation. Additionally, this testing reduced patient fatigue and increased recruitment and ability to give a response as the testing session was usually coupled to a previous appointment and lasted over an hour. If a patient had bilateral DBS, the right STN lead was turned off for the duration of the study visit and returned to the clinical settings following conclusion of the study.

Tests were run at five stimulation settings: first with DBS off, once with each DBS directional contact (A, B, C) turned on individually, and once with the full directional contact on. If the patient’s optimal clinical contact was not directional, then the contact used during testing was the directional contact in closest proximity to the clinical contact. In the case that the patient’s optimal clinical contact had directionality functioning, then the same contact was used during testing. These stimulation parameters were adjusted on the directional lead closest to the patient’s optimal clinical contact with the programming neurologist. A minimum of 2 min was allotted between programming changes.

All sensory testing was completed on the patient’s lower right back, approximately 3 centimeters superior and medial to the posterior iliac crest. Testing was performed on the lower back due to the relatively high incidence of lower back pain in the PD patient population (Broetz et al., 2007), and has previously been described by our group for the testing of sensory thresholds in PD (Belasen et al., 2017). Thermal sensitivity was tested using a Medoc pathway (Ramat Yishai, Israel). The program tested the patient’s cold detection and threshold and heat detection and threshold. Each test was performed twice and results averaged. Mechanical sensitivity was tested using Von Frey fibers (VFF) and pinprick. VFF testing is a well-validated means of mechanical threshold testing and here fibers range from a force of 0.008 to 300 g of pressure (Scitech Korea, Inc., Seoul, South Korea). VFF have shown efficacy in quantifying mechanical sensitivity in previous studies involving both human and animal models (Belasen et al., 2017; Park et al., 2019). The Neuropen is a standardized 10- and 40-g weighted pinprick device. Patients were asked to rate the pain felt after each 10- and 40-g pinprick on a scale of 0–10, with 10 being the highest level of pain. Vibration detection was assessed using a Rydel-Seiffer tuning fork on the spinous process was performed. The patient was to notify the researcher if they could sense the vibration. Pressure pain was measured by a standard pressure gauge device allowing 1–10 kg of pressure to be applied. Patients were asked to notify the researcher when the pressure became uncomfortable and this level was recorded.

Upon completion of sensory testing, the settings which elicited the best sensory response for each patient was determined. This was determined by first evaluating which stimulation parameters elicited the least amount of pain associated with the 40 g weighted pinprick. Then pressure thresholds, sensitivity to VFF, cold/hot pain and cold/hot detection were taken into consideration in successive order. Once the best QST settings were identified, the patient was put under these settings once again, and assessment of the patient’s motor skills was performed using the UPDRS-III motor examination. Once this was complete, the patient’s device was returned to the clinical settings agreed upon by the patient and their treating neurologist. This UPDRS-III score was compared to each patient’s pre-operative UPDRS-III scores as well as their UPDRS-III scores at their optimal clinical setting, which had been previously collected by the patient’s neurologist.

### Statistical Analysis

For demographic and sensory data, repeated measures ANOVAs were performed. Individual statistics were assessed across the five different stimulation settings for each patient and performed for each sensory test. Additional analysis was performed for each sensory test based on the patient’s current clinical settings (directional or non-directional) and the patient’s report of pre-operative pain (pain or no pain). Additionally, a correlation analysis was conducted between stimulation parameters [left sided amplitude, frequency, pulse width (PW), and total electrical energy delivered (TEED)] and outcomes at every contact tested (medial, anterior, lateral, and posterior). A *p*-value less than 0.05 was considered statistically significant.

## Results

### Demographics

In total, nine patients with STN-DBS for PD were enrolled in this study; they had been diagnosed with PD for 13.3 ±1.98 years (mean ±SEM) and were 66.4 ±1.69 years of age (mean ±SEM). Patients underwent sensory testing 12.8 ±1.8 months post-operatively (range 2–19 months). Eight patients were implanted with bilateral STN leads, and one patient was implanted with a unilateral left STN lead. Three patients experienced chronic pain (pain > 3 months) preoperatively, and six patients did not (see Table 1). Patients’ presented for sensory testing on medication. Patients’ levodopa equivalent doses (LEDD) are listed in Table 1. The clinical settings as determined through routine programming as well as contacts used in the study are documented in Table 2. Regarding lead location, the mean ± standard error for each coordinate was 12.06 ± 0.29 mm lateral, 1.4 ± 0.39 mm posterior, and 4.21 ± 0.36 mm inferior. All patients underwent post-operative imaging to confirm electrode placement in the dorsolateral STN. Additionally, the mean amplitude used was 3.4 ± 0.41 mA, frequency was 145.6 ± 5.03 Hz and PW was 70 ± 4.41 μs.

**TABLE 1:** Patient demographics.

Subject number	Sex	Age	Years since diagnosis	History of chronic pain	Location of pain	Clinical contact	Tested contact	KPDPS at testing	NRS at testing	LEDD at time of testing
1	M	59	13	No	−	1	2	11	2	762.5
2	M	64	11	No	−	3	3	0	0	1500
3	M	63	8	No	−	2BC	2	9	1	310
4	M	72	22	Yes	Shoulder	2BC	2	12	1	1312.5
5	M	69	10	Yes	Low back/leg	3AB	3	2	0	1000
6	M	69	8	Yes	Arm	2AB	2	13	0	625
7	M	60	8	No	−	2	2	4	1	925
8	M	75	15	No	−	2AB	2	0	0	250
9	M	67	25	No	−	3− 4 +	3	12	1	1683

**TABLE 2:** Lead location and programming parameters.

Subject number	Coordinates of lead location			Optimal clinical contact	Clinical settings		
	Lateral	Posterior (*anterior)	Inferior		Pulse width (μ s)	Frequency (Hz)	Amplitude (mA)
1	12.19	1.26	2.61	C + 1−	80	130	1.9
2	12.24	0.21*	3.79	C + 3−	140	150	4.5
3	10.06	3.35	4.56	C + 2BC−	60	160	2.8
4	12.01	1.45	5.13	C + 2BC−	90	160	3.7
5	12.75	0.90	4.27	C + 3AB−	60	160	2.8
6	11.65	2.39	4.96	C + 2AB−	70	160	2.5
7	12.34	1.00	5.11	C + 2−	60	130	2.25
8	13.21	0.03	2.35	C + 2AB−	60	140	4.5
9	12.10	2.43	5.10	C + 3-4 +	60	130	4.65

*Represents anterior.

### Quantitative Sensory Testing Results

A repeated measures ANOVA showed that there were no changes seen in post-operative temperature detection or temperature pain thresholds, mechanical sensitivity, weighted pinpricks, vibrational sensitivity, or pressure sensitivity (*p* > 0.1) (see Table 3). A multiple comparisons analysis was also performed to see if there were any significant differences between OFF stimulation vs. omnidirectional, lateral and medial and best setting for sensory outcomes stimulation. Further omnidirectional stimulation settings were compared to lateral, medial and best setting stimulation. Since lead placement varied for patients where anterior lead placement resulted in the “a” contact facing anteriorly while posterior lead placement resulted in the “a” contact facing posteriorly, the anterior contact “a” contact was excluded from the ANOVA and the multiple comparisons analyses.

**TABLE 3:** Results of quantitative sensory testing.

Subject	Clinical contact	Setting	Cold sense (°F)	Warm sense (°F)	Cold pain (°F)	Warm pain (°F)	VFF (g)	Neuropen (10 g)	Neuropen (40 g)	Vibration (yes/no)	Pressure (kg)
1	C + 1−	OFF	−	−	−	−	300.0	0/10	1/10	Yes	5.2
		2 ON	−	−	−	−	1.0	0/10	1/10	Yes	6.1
		2A	−	−	−	−	15.0	0/10	0/10	Yes	5.9
		2B	−	−	−	−	1.4	0/10	1/10	Yes	5.5
		2C	−	−	−	−	2.0	0/10	1/10	Yes	5.5
2	C + 3−	OFF	22.4	42.2	19.8	47.2	4.0	0/10	1/10	No	8.0
		3 ON	25.4	38.2	18.3	44.0	0.6	0/10	1/10	No	4.2
		3A	25.6	42.6	21.1	47.4	2.0	0/10	1/10	No	6.0
		3B	24.6	39.0	20.2	46.4	2.0	0/10	1/10	Yes	6.9
		3C	24.2	38.3	20.1	46.3	1.4	0/10	1/10	Yes	6.5
3	C + 2BC−	OFF	27.8	36.2	0.0	49.7	0.04	0/10	1/10	Yes	6.2
		2 ON	26.0	35.8	0.0	45.3	0.16	0/10	0/10	Yes	6.2
		2A	24.6	35.4	0.0	51.0	0.02	0/10	0/10	Yes	7.0
		2B	23.6	35.3	0.0	51.6	0.04	0/10	0/10	Yes	7.0
		2C	26.9	35.0	0.0	51.2	0.02	0/10	0/10	Yes	8.0
4	C + 2BC−	OFF	24.3	34.3	23.3	36.4	0.4	2/10	5/10	No	1.7
		2 ON	27.1	34.8	26.7	35.7	0.4	3/10	6/10	No	3.0
		2A	29.4	34.7	29.0	35.8	0.4	3/10	6/10	No	2.9
		2B	26.4	34.6	25.9	37.0	1.4	3/10	6/10	No	2.0
		2C	27.8	34.2	25.1	37.2	0.4	3/10	7/10	No	1.8
5	C + 3AB−	OFF	25.1	36.0	0.0	41.4	4.0	0/10	0/10	No	9.2
		3 ON	28.8	34.0	0.0	36.6	0.6	0/10	1/10	No	6.1
		3A	21.1	33.2	0.0	39.4	0.4	0/10	1/10	No	5.0
		3B	28.3	35.0	0.0	38.6	1.4	0/10	1/10	No	7.2
		3C	20.9	34.5	0.0	37.5	1.4	0/10	0/10	No	7.4
6	C + 2AB−	OFF	29.7	35.1	13.9	41.5	1.4	0/10	0/10	Yes	3.5
		2 ON	29.4	34.9	14.2	43.4	8.0	0/10	0/10	No	3.5
		2A	28.6	35.3	16.6	44.3	4.0	2/10	4/10	Yes	5.5
		2B	29.3	34.5	23.3	43.9	1.4	0/10	1/10	Yes	4.6
		2C	27.6	35.4	4.17	43.6	1.4	1/10	3/10	Yes	3.5
7	C + 2−	OFF	26.8	34.8	22.6	36.4	0.04	0/10	1/10	Yes	6.3
		2 ON	29.6	34.5	22.9	37.6	0.16	0/10	1/10	No	4.8
		2A	29.3	34.5	26.5	36.6	0.16	0/10	0/10	No	4.1
		2B	30.3	34.2	24.6	37.7	0.07	0/10	0/10	Yes	5.2
		2C	28.9	34.2	23.3	36.1	0.4	0/10	1/10	No	4.5
8	C + 2AB−	OFF	27.9	34.2	24.1	41.1	4.0	0/10	5/10	No	4.3
		2 ON	22.8	34.3	14.6	39.4	1.0	6/10	8/10	No	3.4
		2A	26.9	34.3	21.0	39.4	0.4	2/10	3/10	No	4.3
		2B	26.8	35.8	20.4	37.9	0.4	1/10	3/10	Yes	3.8
		2C	25.9	35.4	20.9	39.0	4.0	1/10	3/10	No	3.7
9	C + 3−4 +	OFF	24.6	35.3	15.9	38.6	0.16	0/10	0/10	Yes	3
		3 ON	28.1	35.5	0.0	42.6	0.008	0/10	4.5/10	No	3.4
		3A	26.7	36.7	23.5	39.8	1.4	0/10	6/10	Yes	2.9
		3B	26.0	35.6	24.5	39.2	0.008	0/10	3/10	Yes	3.5
		3C	25.3	35.0	21.7	38.9	0.008	0/10	6/10	Yes	3.8

This analysis showed that directional stimulation did not significantly alter patient perceptions of sensory stimulus [cold pain *p* = 0.69, warm pain *p* = 0.99, Von frey fibers (VFF) *p* = 0.09, pin prick *p* = 0.88, vibration *p* = 0.40 or pressure *p* = 0.98]. One patient was removed from the VFF group analysis as the OFF value was greater than 2 standard deviations away from the mean and thus proved to be an outlier. When using VFF, there was a 37.9% increase in sensory threshold using directional stimulation compared to DBS OFF [1.47 ± 0.76 (mean ± SEM)] grams of pressure, although this finding did not reach significance. Individually, two out of nine patients showed this change with medial, 2/9 with lateral, and 3/9 anterior. Two patients showed no change. Further analysis of sensory perception between patients with and without chronic pain showed no significant differences between the two groups in any of the sensory tests we conducted: warm and cold window, warm and cold sense, hot and cold pain, Von Frey Fibers (VFF), 10 and 40 g stimulation direction, vibration stimulation, and pressure stimulation (*p* > 0.05).

Results of the correlation analysis between the stimulation parameters and outcome measures showed that as PW and TEED increased, sensory sensitivity increased when using the anterior, posterior and lateral contact (Table 4). Specifically, when PW increased, the 10 g pin prick (*p* < 0.001; *r* > 0.99) and vibration sensitivity increased (*p* < 0.001; *r* > 0.99) when using the posterior contacts. When TEED was increased, sensitivity to warm detection decreased when using the anterior (*p* = 0.04; *r* = 0.96), lateral (*p* = 0.02; *r* = 0.84) and medial contact (*p* = 0.03; *r* = 0.80). Additionally, increasing TEED caused a decrease in cold detection sensitivity using the medial contact (*p* = 0.03, *r* = −0.8).

**TABLE 4:** Correlation between stimulation parameters and outcome measures.

	Stimulation parameter	Sensory test	*p*-Value	*r*-Value	Sensitivity when stimulation parameters increase
Anterior contact (*n* = 5)	L TEED	Warm detection	0.04	0.96	Decreases
Posterior contact (*n* = 3)	L pulse width	10 g pin prick	<0.001	>0.99	Increases
	L pulse width	Vibration	<0.001	>0.99	Increases
Lateral contact (*n* = 8)	L TEED	Warm detection	0.02	0.84	Decreases
Medial contact (*n* = 8)	L TEED	Warm detection	0.03	0.8	Decreases
	L TEED	Cold detection	0.03	−0.8	Decreases

### Motor Testing Results

In a subset of patients, we were able to assess UPDRS III in ON medication state. We compared the patient’s motor symptoms pre-operatively ON and OFF medications (*n* = 9), ON clinical DBS settings (*n* = 8), and DBS ON that evoked greatest sensory tolerance (*n* = 4). These comparisons showed no statistical differences between DBS ON in the clinical and the best sensory tolerance settings. However, both stimulation of the optimal clinical contact (*p* = 0.0001) and optimal sensory contact (*p* = 0.0058) showed improvement in motor symptoms compared to pre-operatively OFF medication (see Table 5).

**TABLE 5:** Results of UPDRS-III motor assessment.

Subject number	Preop UPDRS-III scores		Optimal clinical settings		Settings during best sensory response	
	Off medication	On medication	Contact	UPDRS-III score ON meds/ON DBS	Contact	UPDRS-III score; ON meds/ON DBS
1	46	9	1−		2	
2	50	28	3−	17	3C	
3	49	35	2−	11	2C	25
4	54	17	2BC−	24	2	37
5	34	15	3AB−	12	3A	22
6	46	30	2AB−	21	2B	
7	44	24	2−	6	2B	
8	48	25	2AB−	18	2A	
9	43	23	3–4 +	37	3B	26

## Discussion

The purpose of this study was to investigate the effects of directional stimulation of STN on sensory perception in Parkinsonian patients. The data collected for this study did not indicate any significant group differences in the tested modalities of sensory perception between directional stimulation and traditional stimulation. However, results of the correlation analysis between the stimulation parameters and outcome measures demonstrated that PW modulated mechanical sensitivity and TEED modulated thermal sensitivity in a predictable manner using certain directional contacts. These preliminary data show promise for improvement in select modalities of pain using directional stimulation.

Previous studies have investigated the effect of both traditional and non-traditional stimulation on sensory perception and pain outcomes. Studies completed with traditional stimulation of omnidirectional leads reported a significant decrease in affective and sensory pain, reduction in pain-induced cerebral activity, an increase in subjective pain thresholds, and a decrease in UPDRS motor scores post-operatively (Dellapina et al., 2012; Pellaprat et al., 2014). Additionally, the use of LFS, compared to both HFS and no stimulation, showed improvements in heat, pressure, and mechanical sensitivity (Belasen et al., 2017). The mechanism behind this relief is not fully understood, but a preliminary study recently found that baseline activity levels of primary somatosensory (SI) may be a promising indicator of whether pain in PD patients will respond well to STN DBS (DiMarzio et al., 2019). Our recent work has also suggested that STN DBS may alter activation patterns in the thalamus and anterior cingulate cortex (ACC) as well as SI.

Several of these functional regions are components of the motor circuit in which increased synchronization of beta band power has been linked to PD (Obeso et al., 2008; Steiner et al., 2019). This motor pathway is well-understood and thought to originate in dorsolateral STN and send output to the globus pallidus pars interna (GPi) which is connected to cortex through the thalamus (Obeso et al., 2008). Similarly, SI and certain nuclei of the thalamus are also components of the somatosensory system’s ascending and descending pathways. As the thalamus is a component of both the sensory and motor pathways, it is possible this overlap could contribute to similar activation patterns between the two. Specifically, the centromedian-parafascicular complex (CM-pf) communicates with both sensory and motor cortices and is thought to parse out persisting multimodal sensory inputs to aid in attending to pain (Ilyas et al., 2019). The most common types of pain in PD patients are neuropathic and nociceptive pain (Pellaprat et al., 2014). Nociceptive pain follows the neospinothalamic ascending tract, which originates in nociceptive neurons in the periphery and ascends the spinal cord to deliver input to both the ventral posterolateral (VPL) and ventral posterior inferior (VPI) thalamic nuclei (Kendroud and Hanna, 2019). From VPL/VPI, nociceptive signals are further processed in SI, producing a painful reaction to noxious stimuli. On the other hand, neuropathic pain is thought to affect interneurons and the descending pathway, in addition to the ascending pathways. Dysfunction in these tracts is thought to alter projections to the thalamus, SI, and the cingulate cortex, resulting in both affective and sensory pain (Colloca et al., 2017).

Although stimulation of the STN has shown efficacy in relieving motor symptoms of PD, a significant limitation of STN DBS is the stimulation of functional areas adjacent to the motor pathway, resulting in a variety of behavioral and sensorimotor impairments such as impulsivity and capsular side effects (Pollo et al., 2014). The use of directional leads, as opposed to conventional omnidirectional leads, has been shown to widen the therapeutic window of DBS, lower the amplitude necessary for treatment, and increase common side effect thresholds (Contarino et al., 2014; Pollo et al., 2014). Thus, it can be suggested that the increased precision and accuracy of directional stimulation has been shown to better target the motor pathway.

Considering the previous efficacy of DBS in modulating sensory perception, this study aimed to investigate whether directional stimulation could further alter sensory perception. These preliminary results implicate that directional leads can modulate components of sensory and thermal perception beyond the improvement seen during omnidirectional stimulation. Specifically, we observed an increase in mechanical sensitivity (as measured by pin prick and vibration testing) when PW was increased in the posterior contact, but not at other contacts. Furthermore, increasing TEED at the anterior, lateral and medial contacts decreased warm detection sensitivity, and decreased cold detection sensitivity at medial contacts. In other words, decreased thermal detection means that patients felt heat at higher temperatures and cold at lower temperatures. As per mechanical sensitivity, this thermal modulation was likewise reproducible and limited to certain directional contacts. The current literature has mixed results regarding STN stimulation on sensory and thermal thresholds, and is limited to studies using omnidirectional leads. For example, studies have demonstrated both an increased thermal detection threshold with STN-DBS (Gierthmuhlen et al., 2010; Maruo et al., 2011), or no change at all (Spielberger et al., 2011). This variability between studies can be attributed to the use of omnidirectional stimulation, differences in testing protocol, and the clinical heterogeneity within PD patients.

Deep brain stimulation surgery is an ever-evolving treatment which has shown continuous improvement in treating PD with the development of directional leads within the last decade. These multi-contact leads have shown promise in treating not only the primary motor symptoms of PD but also the prevalent non-motor components of the disease. This study is the first to investigate the effect of directional stimulation on sensory perception in patients diagnosed with PD and receiving STN stimulation. Our findings suggest that directional stimulation can modulate certain modalities of sensory perception in addition to motor symptom relief.

We are aware that there are several limitations of this study. This includes the subjective nature of QST, in which pain scales may not be held constant across patients. Additionally, patients were aware of the stimulation parameters being tested during the study. The number of patients studied was small, but is consistent with other dDBS studies at this time. Both lead location and programming parameters, while variable between patients, did not demonstrate any outliers that were consistent with individual variation in DBS among PD patients. UPDRS-III scores at the patient’s optimal clinical contact and without stimulation (medication on and off) were collected during the previous programming sessions and pre-operatively respectively, such that all UPDRS scores for a given patient were not collected on the same day. It is the goal of the authors that future studies include comparisons between the relief of both pain and motor symptoms in a larger population of PD patients whose clinical settings are set to either omnidirectional or directional stimulation. Further investigation with a larger patient population will allow for further comparative sensory testing in patients with and without chronic pain that can identify between-group differences. Additionally, analyzing changes in quality of life measures before and after surgery could elucidate the effect of directional leads on quality of life for PD pain patients. Studying different frequencies may also elucidate the role of directionality versus frequency in sensory changes as it has been hypothesized that frequency may play a larger role in this. However, it has been shown that patients with tremor predominant PD cannot tolerate lower frequencies for motor symptom control which led us to investigate directionality as an alternative approach in this current study (Xie et al., 2015; Belasen et al., 2017; Oza et al., 2018).

## Conclusion

This study aimed to determine the effect of directional stimulation of STN on sensory perception in patients with PD. Directional stimulation resulted in increased thresholds to mechanical stimulation, specifically increasing PW increased pin prick and vibration sensitivity when using the posterior contact. Furthermore, decreased thermal detection sensitivity was seen with increased TEED in specific directional contacts. These findings expand the current therapeutic knowledge of directional DBS beyond the relief of motor symptoms and provide promise for the use of directional leads to modulate certain aspects of sensory perception.

## Data Availability Statement

The datasets generated for this study are available on request to the corresponding author.

## Ethics Statement

The studies involving human participants were reviewed and approved by the Institutional Review Board at Albany Medical College. The patients/participants provided their written informed consent to participate in this study.

## Author Contributions

SS helped to organize the project, obtain and analyze the data, write and review the manuscript. OK helped to analyze the data, write and review the manuscript. MD helped to organize the project, obtain and analyze the data, write and review the manuscript. MS aided in writing, reviewing, and critiquing the manuscript. LW helped to obtain and analyze the data and wrote the manuscript. MG obtained the data and reviewed and critiqued the manuscript. JD, EH, and OA provided intellectual aid, reviewed, and critiqued the manuscript. JP developed the study, organized the project, wrote, reviewed, and critiqued the manuscript.

## Conflict of Interest

JP is a consultant for Boston Scientific, Nevro, TerSera, and Abbott and receives grant support from Medtronic, Boston Scientific, Abbott, Nevro, TerSera, GE Global Research, SBIR 1R43NS107076-01A1, NIH 2R01CA166379-06, and NIH U44NS115111. She is medical advisor for Aim Medical Robotics and Karuna and has stock equity. The remaining authors declare that the research was conducted in the absence of any commercial or financial relationships that could be construed as a potential conflict of interest.

## Footnotes

1 https://www.parkinson.org/Understanding-Parkinsons/Statistics
